# MRI findings in children with migraine or tension-type headache

**DOI:** 10.1186/s12887-023-04264-y

**Published:** 2023-08-30

**Authors:** Rabia Tütüncü Toker, Ilknur Ozdeniz Mutlucan, Cigdem Tanrıverdi, Aylin Bican Demir

**Affiliations:** 1 Department of Pediatrics, Division of Pediatric Neurology, University of Health Sciences, Bursa City Training and Research Hospital, Doganköy, Nilüfer, Bursa, +90 506 366, 3796 Turkey; 2 Department of Radiology, Ilknur Ozdeniz Mutlucan, University of Health Sciences, Bursa City Training and Research Hospital, Radiologist, Bursa, Turkey; 3 Child and Adolescent Psychiatrist, Nev Hospital, Bursa, Turkey; 4 Department of Neurology, Bursa Uludag University, Bursa, Turkey

**Keywords:** Headache, Primary, Secondary, Neuroimaging, Mrı, Papilledema

## Abstract

**Purpose:**

Migraine and tension-type headache are common primary headaches in children. There is a risk of developing secondary headache in children. The current study was aimed to evaluate magnetic resonance imaging findings (MRI) in children with migraine or tension-type headache.

**Methods:**

The study was planned in children with migraine or tension-type headaches who have been followed up in the pediatric neurology outpatient clinic with regular office visits for at least two years and had neuroimaging in the last year.

**Results:**

280 patients (187 female patients) datas were studied. 91 (61 female patients) were followed up with the diagnosis of migraine and 189 (126 female patients) with the diagnosis of tension-type headaches. The age of patients was found to be 13.1 ± 3.4 years. Brain tumor was found in one child with tension-type headache who had papilledema. Incidental MRI findings found 7.7% and 12.7% in migraine and tension-type headache, respectively. MRI findings in the study were arachnoid cyst (14), pituitary adenoma (6), mega cisterna magna (6), pineal cyst (3), non-specific gliosis (2) and tumor (1).

**Conclusion:**

Arachnoid cysts were found incidental as the most common MRI finding in children with migraine or tension-type headache. The rare life-threatening secondary headache may develop in children. The fundus examination as a complement to the neurological examination can be useful for requesting MRI.

## What is known

- Headache in childhood is an important cause of emergency and outpatient referral for pediatricians.
- Secondary headaches can be caused by life-threatening causes such as tumors.
- Red flags have been developed for secondary headaches.
- MRI, which is an expensive neuroimaging, is widely used in the diagnosis of headache.

## What is new

- Children with known migraine and tension-type headaches have repeated MRI even in emergency room or outpatient clinics.
- Asymptomatic arachnoid cysts were found most frequently in MRIs of children with migraine or tension-type headache.
- Papilledema was found to be the determinant of secondary headache risk in children with migraine or tension-type headache.

## Purpose

Headache is common complaint in children [1]. It is important to evaluate the headache correctly in children, which is worrisome for the family. Although primary headache is more frequent in children as in adults, the possibility of secondary headache is frightening for families and even physicians because of underlying disease such as central nervous system infection, brain tumors or vascular disorders. Diagnostic criteria for primary headache have been developed and the International Classification of Headache Disorders of the International Headache Society guidelines have been published [2]. Migraine and tension-type headaches are main primary headaches of childhood. The role of Magnetic Resonance Imaging (MRI) in headache is controversial. MRI for each headache is costly, so studies are underway to understand whether a headache is primary or secondary without using tools of neuroimaging. Warning red flags have been developed for neuroimaging in secondary headaches. Data on the incidence and prevalence of secondary headaches, as well as the sensitivity, specificity, and predictive value of red flags for secondary headaches are discussed [3, 4]. Evaluation of pediatric patients with headaches is based on an adequate medical history as a first step and focused on looking for so-called red flags, warning signs suggestive of secondary headache. Among these red flags, some are considered to be at higher risk, such as pain that wakes the child from sleep or that occurs upon awakening, worsening of headaches with supine and/or straining, coughing [5]. Red flags developed to differentiate primary and secondary headaches are of great importance. However, what could be the symptom or finding that could be a clue in case of secondary headache that may develop in a patient who has already been diagnosed with primary headache. There is always a risk that a secondary headache may develop over time in children with primary headache. Moreover, since it is a diagnosis of primary headache, the attention of the clinician or caregiver may make it difficult to understand the headache feature and may lead to ignoring potentially dangerous etiologies. However, the clinician’s approach to headache may also be more rigorous or anxious, resulting in the use of costly MRI. The aim of this study is to evaluate MRI findings in children with known as migraine or tension-type headaches.

## Methods

The study was conducted in a tertiary healthcare center, including patients with migraine or tension-type headaches under the age of 18, who have been followed up in the pediatric neurology outpatient clinic with regular office visits for at least two years and had MRI in the last year. The International Classification of Headache Disorders criteria of the International Headache Society were taken as the diagnostic criteria for migraine and tension-type headaches [2]. Demographic information such as age, gender and findings of MRI were taken as data. The request for MRI were divided into 3 groups. These groups are;

Group 1: Patients with abnormal findings in neurological examination (papilledema, focal neurologic finding or deficit),

Group 2: Patients with red flags (systemic symptoms, acute onset, occipital zone, precipitated by Vasalva, positional, progressive, < 6 years old).

Group 3: Patients with no abnormal neurologic findings or with no red flags.

Ethics committee approval was obtained from the local ethics committee for the study.

### Statistical analysis

SPSS 23.0 statistical package program was used in the analysis of the data. Descriptive statistics of evaluation results; number and percentage for categorical variables; are given as mean and standard deviation (ss) for metric variables.The conformity of the measurement data to the normal distribution was examined with the One-sample Kolmogorov Smirnov Test. Comparisons of the measurement data of two independent groups; Since the normal distribution condition was not met, the Mann-Whitney U test was used.Chi-square test was used to analyze the differences between categorical ratios.Statistical significance level was accepted as p < 0.05.

## Results

It was found that 840 children and adolescents with the diagnosis of migraine or tension-type headaches had regular office visits. The average follow-up period of the patients is 32 months (min: 25, max: 47 months). Of these patients, 280 patients (187 female patients) were identified from the files of the patients who had undergone MRI in the last year. Of the patients, 91 (% 61 female) were with migraine and 189 (%67 female) with tension-type headaches. Female gender (67%, 66.7%) was dominant in both groups, and there was no difference between groups (p > 0.05). The age of patients with migraine was found to be 13.6 ± 3.2 (Table 1).

**Table 1:** Demographic characteristics patients are shown

	Migraine (n = 91)	Tension-type headache (n = 189)	Totals (n = 280)	P value
**Age (mean ± SS)**	13.6 ± 3.2	12.8 ± 3.5	13.1 ± 3.4	0.100
**Gender n (%)**				
**Female**	61 (67.0)	126 (66.7)	187 (66.8)	0.988
**Male**	30 (33.0)	63 (33.3)	93 (33.2)	

When the MRI requests of the patients were evaluated, there is no abnormal neurologic findings or no red flags in 40.7% of the patients with migraine and 68.8% (n: 130) of the patients with tension-type headache (Table 2). Three of the children with migraine and one child with tension-type headache had abnormal neurological examination findings. These findings were papilledema in one child with tension-type headache and one had temporary vision loss in one child with migraine, and two had one-sided temporary paralysis. Although children with migraine and neurological symptoms did not have their first appointment and these findings were known to be temporary, MRIs were requested by the doctor in the emergency rooms they applied to.

**Table 2:** Comparison of magnetic resonance imaging findings of children with migraine and tension-type headache

	Migraine (n = 91) n (%)	Tension-type headache (n = 189) n (%)	Totals (n = 280) n (%)	P value
**Reasons for requesting MRI**				
Group 1 (abnormal neurological examination)	3 (3.3)	1 (0.5)	4 (1.4)	< 0,001
Group 2 (red flags)	51 (56.0)	58 (30.7)	109 (38.9)	
Group 3	37 (40.7)	130 (68.8)	167 (59.6)	
**MRI Findings**				
Normal	84 (92.3)	164 (86.8)	248 (88.6)	p value cannot be given
Incidental	7 (7.7)	24 (12.7)	31 (11)	
Abnormal	null	1 (0.5)	1 (0.4)	
**MRI Request Site**				
Outpatient Clinic	73 (80.2)	150 (79.4)	223 (79.6)	0,942
Emergency Room	18 (19.8)	39 (20.6)	57 (20.4)	

There was no abnormal MRI report in migraine patient. One (0.5%) patient with tension-type headache had abnormal MRI report. The patient has been followed up with tension-type headache for about 3 years, had a normal MRI 4 years ago This patient was a 12-year-old boy with a tumor located in the unilateral posterior thalamus.The tumor had a mass effect. The patient had headache, vomiting and papilledema on neurological examination. Incidental MRI findings found 7.7% and 12.7% in migraine and tension-type headache, respectively. Arachnoid cyst was found to be the most common MRI finding (Table 3). The percentage of patients with arachnoid cyst is found 5%.

**Table 3:** MRI findings of pediatric patients with migraine and tension-type headache are shown

Neuroimaging Findings		n
Incidental (n:31)	Arachnoid cyst	14
	Mega cisterna magna	6
	Pituitary adenoma / Empty sella (partial)	6
	Pineal cyst	3
	Non-specific gliosis	2
Abnormal (n:1)	Tumor	1

## Discussion

**Fig. 1:**
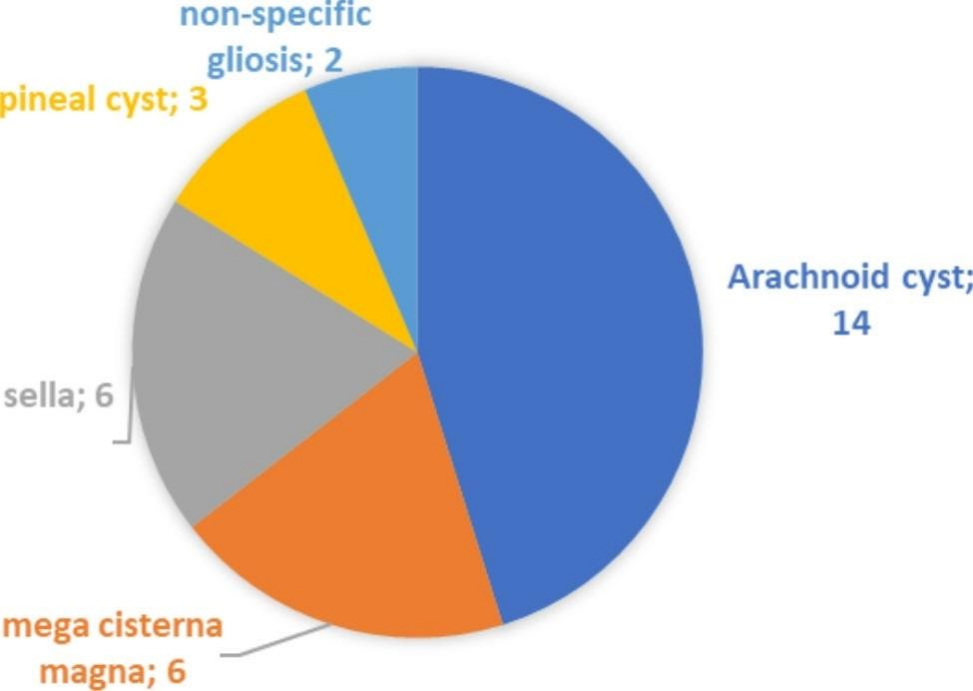
Incidental MRI findings of patients are shown

In this study, there were no abnormal MRI reports in migraine, and only one abnormal MRI report was in tension-type headache. Similarly in the literature, Tsushima et al. reported that repeated neuroimaging was unremarkable in adults with chronic headache and had no features in previous neuroimaging, and neuroimaging was unnecessary in patients with normal neurological examination [6]. On the other hand, it has been reported in publications that when no pathology is detected in neuroimaging, this will reduce the anxiety of the family and they can better cope with the headache [7]. In our study, MRI was performed in 96.7% of migraine patients and 99.5% of tension-type headache, although the neurological examination was normal, moreover, almost one-fifth of these MRI’s were performed in the emergency room. This raises the question of when MRI should be performed in children with primary headache. This is a difficult and complex question to answer. Red flags, neurological examination, the child’s assessment and expression of the current headache, the approach of the physician evaluating the child, and as a result of all these, it becomes difficult to decide whether neuroimaging is needed. In our study, when MRI findings were evaluated, a tumor was found as an abnormal MRI finding in a patient with tension-type headache in the presence of papilledema, which is a neurological examination finding. Arachnoid cysts were found incidental as the most common MRI finding in this study(Fig. 1). The increasing use of MRI has led to more frequent diagnosis of arachnoid cysts. The percentage of patients with arachnoid cyst is found 5%. Al-Holou et al. reported that arachnoid cysts remained clinical and imaging stable with increasing age and did not require any intervention in their study in a large series of pediatric populations [8]. On the other hand, surgery has been found to be beneficial when arachnoid cysts are symptomatic [9], and it has been reported that surgically treated arachnoid cysts have headache with signs of increased intracranial pressure [10]. In our study, arachnoid cysts were accepted as incidental because they did not show any signs of increased intracranial pressure in their current state. In this study, fundus examination, which is an important complementary part of the neurological examination, was the most important clue for secondary headache. The only abnormal MRI finding was found in a patient with papilledema. It is a known fact that one of the causes of papilledema is space-occupying lesions in the brain such as intracranial tumors [11–13].

In our study, almost 20% of MRIs were taken in the emergency room. This situation forces us to consider for what indication the high-cost MRI was taken in the emergency room. Moreover, no reason for MRI was found for patients in group 3 who had no abnormal neurological examination findings or red flags. We can only assume that there may be physician or patient or family concerns, but due to the retrospective nature of our study, we cannot give an exact answer to this question. The relationship has been reported between childhood headache and the presence of emotional and behavioral symptoms. Children with headache may be more anxious and may describe the headache character differently than it actually is. This may affect the evaluation of families and physicians. Migraine, which is very common; it is known that psychiatric diagnoses such as depression, anxiety and post-traumatic stress disorder often accompany them [14]. The sociocultural and economic levels of those describing migraine-type headache were found to be high. In addition, it has been found that most of these people have a high desire for success, perfectionist, prescriptive, meticulous and anxious. In many studies conducted after 1990, it has been determined that individuals with migraine go to centers specialized for headaches they have experienced due to these personality traits [15]. Early onset of migraine in children and severity of migraine were associated with higher levels of family reunification [16]. Considering the family history of migraine sufferers, the fact that both the children with migraine and their families are perfectionist, prescriptive, meticulous and anxious, may have led physicians to need neuroimaging as an additional examination, even if they do not have neurological symptoms. Since our study was designed retrospectively, the reasons for MRI of the patients in group 3 could not be explained. The limiting factor of this study is its retrospective design. It seems that we need to understand more about recurrent MRI requests in primary headaches. There is a need for future prospective studies to understand the attitude of physicians in emergency rooms or outpatient clinic We hope that future prospective studies will define the reasons that push the physician to request MRI in children who do not have abnormal neurological findings or red flags.

This study suggests that physician should spare time for fundus examination for children with primary headache during their regular office visits or in emergency rooms where they apply with headache.

## Conclusion

This study; shows that the most important indication for MRI in children with primary headache is papilledema. It would be more appropriate to consider neuroimaging in the presence of abnormal neurological examination findings in children with primary headache.

## Data Availability

The datasets used and/or analyzed during the current study are available from the corresponding author upon reasonable request.

## References

[1] Blume HK (2017). Childhood headache: a brief review. Pediatr Ann.

[2] Headache Classification Committee of the International Headache Society (IHS) (2018). The International classification of Headache Disorders, 3rd edition. Cephalalgia.

[3] Do TP, Remmers A, Schytz HW (2019). Red and orange flags for secondary headaches in clinical practice: SNNOOP10 list. Neurology.

[4] Dao JM, Qubty W (2018). Headache diagnosis in children and adolescents. Curr Pain Headache Rep.

[5] Prezioso G, Suppiej A, Alberghini V et al. Pediatric Headache in Primary Care and Emergency Departments: Consensus with RAND/UCLA Method. Life (Basel). 2022;12(2):142. Published 2022 Jan 19. 10.3390/life12020142. 10.3390/life12020142 PMC8877535 35207430

[6] Tsushima Y, Endo K (2005). MR imaging in the evaluation of chronic or recurrent headache. Radiology.

[7] Pavone P, Conti I, Le Pira A, Pavone L, Verrotti A, Ruggieri M (2011). Primary headache: role of investigations in a cohort of young children and adolescents. Pediatr Int.

[8] Al-Holou WN, Yew AY, Boomsaad ZE, Garton HJ, Muraszko KM, Maher CO (2010). Prevalence and natural history of arachnoid cysts in children. J Neurosurg Pediatr.

[9] Deopujari CE, Shaikh ST, Karmarkar VS, Sudke AY, Mohanty CB, Biyani NK (2021). Experience with management of Intracranial Arachnoid Cysts. J Neurol Surg A Cent Eur Neurosurg.

[10] Pradilla G, Jallo G (2007). Arachnoid cysts: case series and review of the literature. Neurosurg Focus.

[11] Xie JS, Donaldson L, Margolin E, Papilledema (2022). A review of etiology, pathophysiology, diagnosis, and management. Surv Ophthalmol.

[12] Hyde RA, Mocan MC, Sheth U, Kaufman LM (2019). Evaluation of the underlying causes of papilledema in children. Can J Ophthalmol.

[13] Crum OM, Kilgore KP, Sharma R (2020). Etiology of Papilledema in patients in the Eye Clinic setting. JAMA Netw Open.

[14] Minen MT, De Begasse O, Van Kroon A (2016). Migraine and its psychiatric comorbidities. J Neurol Neurosurg Psychiatry.

[15] Eidlitz-Markus T, Haimi-Cohen Y, Zeharia A (2015). Association of age at onset of migraine with family history of migraine in children attending a pediatric headache clinic: a retrospective cohort study. Cephalalgia.

[16] Stewart WF, Bigal ME, Kolodner K, Dowson A, Liberman JN, Lipton RB (2006). Familial risk of migraine: variation by proband age at onset and headache severity. Neurology.

